# The Tonic Effects of the Hypophosphites

**Published:** 1913-04

**Authors:** Alfred S. Gubb

**Affiliations:** M. D., L. R. C. P. Lond., M. R. C. S. Eng., D. P. H., Etc., Aix-Le-Bains, Savoie, France


### Abstracts and Selections.

### The Tonic Effects of the Hypophosphites

BY ALFRED S. GUBB, M. D., L. R. C. P. LOND., M. R. C. S. ENG., D. P. H., ETC., AIX-LE-BAINS, SAVOIE, FRANCE.

The choice of a tonic for the young is determined by special considerations, because their requirements differ in several important respects from those of adults. The interference with nutrition that arises from acute pyrexial infections is attended by particularly serious consequences in the young, because their growing tissues are less stable and their mineral constituents are less

"fixed," so that the disintegrating effects of exaggerated oxidation are much more rapidly produced.

The requirements of the youthful organism in the matter of inorganic salts are much greater than those of later life, because not only has the ordinary wear and tear to be provided for, but also the demands of growth. Acute disease, as we know, is characterized by more rapid oxidation and exaggerated metabolism, and the latter, in turn, is associated with wasteful leakage of the mineral constituents of the tissues.

Depleted of their mineral constituents, the tissues protect themselves as best they can by drawing on the organic reserves, especially the bones, so that pyrexia not only entails arrest of development, but also of positive denutrition.

Then, too, the blood pressure in the young is normally low compared with that of the adult, so that the depression resulting from the ravages of disease is particularly well marked.

Apart from acute disease, children suffer from a series of affections associated with chronic malnutrition. Dyspepsia, rickets, scurvy-rickets, etc., are all characterized by impoverishment of the tissues, so that, although less rapidly induced, the constitutional changes are, roughly speaking, the same. The consequences, however, are more serious, because they are more protracted. The lack of mineral salts in general and the compounds of phosphorus with the earthy bases in particular, leave the skeleton abnormally plastic, so that the traction of the muscles produces deformities of the thorax and limbs, which deformities are likely to become permanent unless speedily remedied.

The recognition of this condition affords the requisite data for the choice of the most suitable tonic. The weakly infant is the victim of more or less pronounced demineralization of the tissues, and the indication is that it should be provided with the material for remineralization in the form best adapted for immediate assimilation. At the same time, tone must be restored to the vascular system and to the muscles, in order to secure the performance of the vital functions.

Now, the salts of which the youthful organism stands most in need are compounds of phosphorus, and, as its capacity for assimilation is limited in regard to any particular salt, the phosphorus must be combined with various bases. Of all the phosphorus compounds, the hypophosphites are recognized to be the most readily utilized by the tissues; hence a compound syrup of these salts will fulfill every indication.

The vasotonic element—strychnine—may advantageously be associated with these salts, as it is in Fellows' syrup of the hypophosphites.

Fellows' syrup is less a medicine than a food, just as salt is a food and not a mere condiment. Without salt the tissues are unable to assimilate and to retain the elements indispensable to their nutrition and growth. Under the influence of the hypophosphites they not only gain in weight, but also in density, owing to the fixation of inorganic salts, and so the balance of nutrition is restored,—*The Medical World.*

